# Donor Derivative Incorporation: An Effective Strategy toward High Performance All‐Small‐Molecule Ternary Organic Solar Cells

**DOI:** 10.1002/advs.201901613

**Published:** 2019-09-04

**Authors:** Hua Tang, Tongle Xu, Cenqi Yan, Jie Gao, Hang Yin, Jie Lv, Ranbir Singh, Manish Kumar, Tainan Duan, Zhipeng Kan, Shirong Lu, Gang Li

**Affiliations:** ^1^ Chongqing Institute of Green and Intelligent Technology Chinese Academy of Sciences Chongqing 400714 China; ^2^ University of Chinese Academy of Sciences Beijing 100049 China; ^3^ Department of Electronic and Information Engineering The Hong Kong Polytechnic University Hong Hum Kowloon Hong Kong China; ^4^ Department of Energy & Materials Engineering Dongguk University Seoul 04620 Republic of Korea; ^5^ Pohang Accelerator Laboratory Pohang University of Science and Technology Pohang 37673 Republic of Korea

**Keywords:** morphology, organic solar cells, small molecules, structural similarity, thick films

## Abstract

Thick‐film all‐small‐molecule (ASM) organic solar cells (OSCs) are preferred for large‐scale fabrication with printing techniques due to the distinct advantages of monodispersion, easy purification, and negligible batch‐to‐batch variation. However, ASM OSCs are typically constrained by the morphology aspect to achieve high efficiency and maintain thick film simultaneously. Specifically, synchronously manipulating crystallinity, domain size, and phase segregation to a suitable level are extremely challenging. Herein, a derivative of benzodithiophene terthiophene rhodanine (BTR) (a successful small molecule donor for thick‐film OSCs), namely, BTR‐OH, is synthesized with similar chemical structure and absorption but less crystallinity relative to BTR, and is employed as a third component to construct BTR:BTR‐OH:PC_71_BM ternary devices. The power conversion efficiency (PCE) of 10.14% and fill factor (FF) of 74.2% are successfully obtained in ≈300 nm OSC, which outperforms BTR:PC_71_BM (9.05% and 69.6%) and BTR‐OH:PC_71_BM (8.00% and 65.3%) counterparts, and stands among the top values for thick‐film ASM OSCs. The performance enhancement results from the enhanced absorption, suppressed bimolecular/trap–assisted recombination, improved charge extraction, optimized domain size, and suitable crystallinity. These findings demonstrate that the donor derivative featuring similar chemical structure but different crystallinity provides a promising third component guideline for high‐performance ternary ASM OSCs.

## Graphical abstract

**A donor derivative is incorporated in benzodithiophene terthiophene rhodanine (BTR)‐based thick‐film all‐small‐molecule (ASM) organic solar cells (OSC)**, which achieves power conversion efficiency of 10.14% and fill factor of 74.2%, outperforms its binary counterparts, and stands the record value for thick‐film dual‐donor ternary ASM OSCs. The results demonstrate that the donor derivative is a promising third component to boost the performance of ASM OSCs.

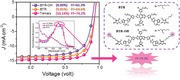

## 1 Introduction

Solution‐processable all‐small‐molecule (ASM) organic solar cells (OSCs) consisting of a small‐molecule donor and a small‐molecule acceptor are currently attracting enormous attentions due to their distinct advantages such as monodispersion, easy purification, and scalability with negligible batch‐to‐batch variation.^1^, ^2^, ^3^, ^4^, ^5^ Tremendous progress has been made in the past years on rational molecule design, device engineering, and interface modification, leading to over 10% power conversion efficiencies (PCEs) in ASM OSCs with fullerene derivatives as the electron acceptor.^6^, ^7^, ^8^, ^9^ However, the difficulties of controlling the morphology (e.g., crystallinity and domain size) of active‐layer constrained the development of ASM OSCs.^10^, ^11^, ^12^ Furthermore, the device performance of ASM OSCs is often sensitive to the film thickness of ≈100 nm in most reports, which hinders the future high‐throughput device fabrication processing like roll‐to‐roll and ink jet printings.^13^, ^14^, ^15^ Thus, it is worth finding an effective method to tune the active‐layer morphology, and attain high efficiency with thick active layers. Benzodithiophene terthiophene rhodanine (BTR) stands out as an excellent thick‐film OSC material with respectable (while not enough) efficiency of over 9% pairing with fullerene acceptor. Finding effective methods to further enhancing the BTR based thick‐film OSC device efficiency is expected to be an important and viable way toward manufacture friendly high performance OSCs.

Ternary OSCs, which consist of a donor, an acceptor, and a third component, have the potential to outperform their binary single‐junction counterpart. In such devices, the third component usually plays the role of broadening the absorption spectra, helping charge transfer and/or transport, reducing carrier recombination, and optimizing the active‐layer morphology.^16^, ^17^, ^18^, ^19^, ^20^, ^21^, ^22^, ^23^, ^24^, ^25^, ^26^, ^27^ Achieving good morphology in ternary bulk‐heterojunction (BHJ) is a challenging task, and the success of utilizing structural similar donors in ternary BHJ OSC represents an important concept in the field. One of us, Li, and Yang et al. demonstrated in 2015 that structural compatible polymer donors (which have similar chemical building block) with complementary absorption spectrum could coexist harmoniously, resulting in less interference when forming the morphology of the active layer and maintain similar molecular packing as well as orientation, which finally enabled higher PCE of 8.7% for ternary OSC (PTB7: PBDTT‐SeDPP: PC_71_BM = 0.5:0.5:2 in wt%) than that of its corresponding binary OSC.^28^ The concept was recently further extended to dual NFA acceptors based ternary OSC, where Liu et al. utilized two structure‐similar and absorption‐similar acceptors (ITCPTC and MeIC) to fine tune the crystallinity and the domain size of PM6:ITCPIC:MeIC blend. When the load of MeIC reached 40 wt% with respect to ITCPTC in the active layer, the corresponding ternary OSC exhibited optimal crystallinity with coherence length of 18.5 Å and suitable domain size of 23.5 nm, achieving higher PCE of 14.13% and fill factor (FF) of 78.2% than those of the binary counterparts.^29^ In addition to structural similarity concept, OSC material crystallinity is another useful parameter for designing ternary OSCs. Nicola et al. reported a host amorphous polymer donor with efficient charge separation ability (PTB7) cooperates with another more crystalline polymer donor (Si‐PCPDTBT) that facilitates the charge transport, meanwhile, suppresses the charge recombination, leading to a remarkable FF of 77% and enhanced PCE of 8.60% for the dual‐polymeric‐donor ternary OSC.^30^

To our knowledge, the above‐mentioned simple yet effective strategy of employing a structure similar third component has never been demonstrated in solution‐processed all‐small‐molecule OSCs. Herein, by introducing hydroxyl into the rhodamine group of BTR, we intentionally synthesized a small‐molecule donor derivative of BTR, BTR‐OH, with similar chemical structure and absorption profiles, yet weaker crystallinity.^31^ We added BTR‐OH into the BTR:PC_71_BM binary host system to construct ternary ASM OSC. The BTR‐OH effectively increased the absorption of the ternary blend films in the region from 400 to 600 nm. Upon adding the less crystalline BTR‐OH, the phase segregation in the active layer was slightly weakened while the high hole mobility and high PCE at thick‐film were retained. This shows that BTR‐OH acts as an excellent morphology adjuster to fine tune active layer morphology, leading to enhanced exciton dissociation and charge collection, reduced charge recombination, and suitable domain size of ≈20 nm. The corresponding ternary ASM OSCs achieved a high PCE of 10.14% and a FF of 74.2% at the film thickness of ≈300 nm, outcompeting BTR:PC_71_BM counterparts (PCE of 9.05% and FF of 69.6%). To the best of our knowledge, the PCE of 10.14% is the highest reported value for thick‐film ternary ASM OSC using two small molecule donors, which provides new insight into ternary ASM OSC design.^13^, ^17^, ^31^, ^32^, ^33^

## 2 Results and Discussion

**Figure 1:**
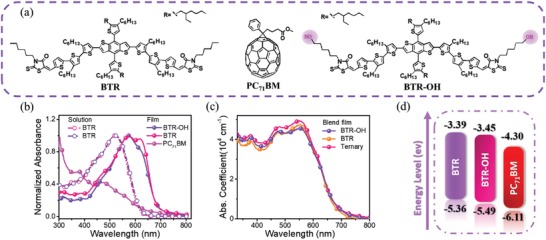
a) Molecular structures of the small‐molecule donors (BTR and BTR‐OH), and fullerene acceptor (PC_71_BM). b) Normalized UV–vis absorbance spectra of BTR, BTR‐OH in thin‐films and solutions and PC_71_BM neat films. c) The absorption coefficient spectra of BTR‐OH:PC_71_BM, BTR:PC_71_BM, and ternary films. d) Energy level diagram of BTR, BTR‐OH, and PC_71_BM.

**Figure** **1**a illustrates the molecular structures of BTR, BTR‐OH, and PC_71_BM. The difference between BTR and BTR‐OH lies in the hydroxyl substitution in the BTR's rhodamine end group. Figure 1b shows that the solution and neat film absorbance of BTR‐OH resembles that of BTR. The absorption maxima of BTR and BTR‐OH films are at 574 and 577 nm, which are both redshifted by 55 nm relative to those in solution, implying the presence of strong intermolecular interaction in the solid film. These donors both exhibited absorption onset of 680 nm, which accounts for the optical bandgap of 1.82 eV and well complements the absorption of PC_71_BM. Comparing these two donor films, BTR clearly has stronger crystallinity, evidenced by the vibronic peak at ≈630 nm, at which BTR‐OH has just an absorption shoulder. Among the films with different weight ratio, the BTR: BTR‐OH: PC_71_BM (0.8: 0.2: 1, w/w) blend film exhibits the highest maximum extinction coefficients at 552 nm (Figure 1c), which is potentially advantageous to the harvest of photons. Figure 1d presents the energy level diagrams of BTR, BTR‐OH, and PC_71_BM, which was determined by cyclic voltammetry method (Figure S1, Supporting Information). BTR‐OH displays the lowest unoccupied molecular orbital (LUMO) level of −3.45 eV, slightly lower/deeper than that of BTR (−3.39 eV). The highest occupied molecular orbital (HOMO) level of BTR‐OH (−5.49 eV) lies between those of BTR (−5.36 eV) and PC_71_BM (−6.11 eV), which forms an ideal energy cascade favorable for charge separation.

**Figure 2:**
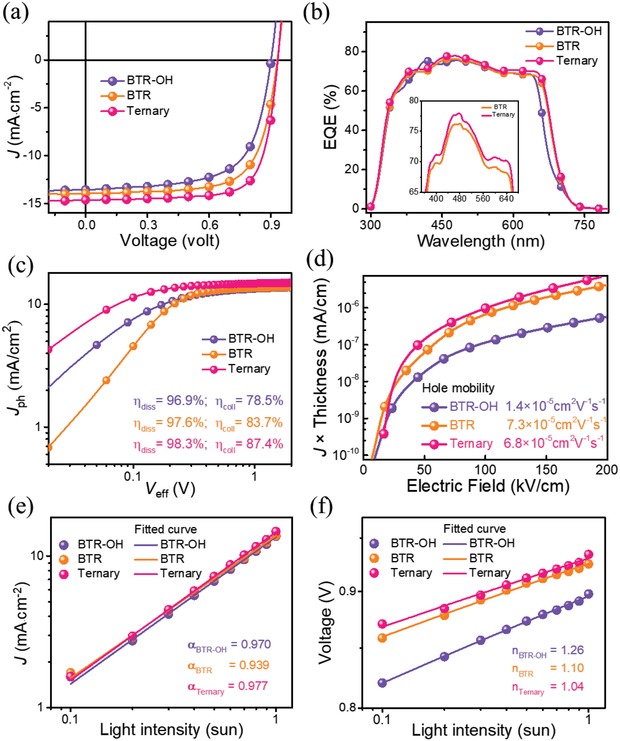
a) *J*–*V* characteristics, b) EQE spectra, and c) Photocurrent density (*J*_ph_) as a function of effective voltage (*V*_eff_) curves of the optimized binary and ternary OSCs. d) Hole Mobilities. e) *J*_SC_ and f) *V*_OC_ as a function of incident light intensity of the optimized binary and ternary devices.

Thick‐film OSCs in bulk heterojunction (BHJ) were fabricated with a conventional device architecture of ITO/PEDOT:PSS/active layer/phenyl(2‐naphthyl)diphenylphosphine oxide (DPO)/Ag. The weight ratio of donor and acceptor was fixed at 1:1, while the ratio of BTR:BTR‐OH was varied from 1:0 to 0:1. The best performing device was obtained when the ratio of BTR‐OH was at 0.8:0.2. **Table** **1** summarizes the figure of merits of the optimized OSCs with BTR:PC_71_BM, BTR‐OH:PC_71_BM, and BTR:BTR‐OH:PC_71_BM as the active layer, all measured under simulated AM1.5G irradiation (100 mW cm^−2^) condition. **Figure** **2**a depicts the current density–voltage (*J*–*V*) characteristics of the optimized OSCs.

**Table 1:** Photovoltaic performance of optimized OSCs based on BTR: PC_71_BM, BTR‐OH: PC_71_BM, and BTR:BTR‐OH: PC_71_BM under simulated AM1.5G illumination (100 mW cm^−2^)

D1:D2:A^a)^	Film thickness [nm]	SVA^b)^ [s]	*V*_OC_ [V]	*J*_SC_ [mA cm^−2^]	*Calc. J*_SC_ [mA cm^−2^]	FF [%]	Avg. PCE^c)^ [%]	Max. PCE [%]
1:0:1	≈300	25	0.93	13.95	13.69	69.6	8.93	9.05
0:1:1	≈280	40	0.90	13.56	13.15	65.3	7.85	8.00
0.8:0.2:1	≈300	35	0.93	14.62	14.03	74.2	9.98	10.14

^a)^ D1 = BTR, D2 = BDT‐OH, and A = PC_71_BM

^b)^ Solvent vapor annealing (SVA). The solvent used in this work was DCM

^c)^ Average values were obtained from 20 devices.

The BTR:PC_71_BM‐based device showed a high PCE of 9.05% with a *V*_OC_ of 0.93 V, a *J*_SC_ of 13.95 mA cm^−2^, and a FF of 69.6%. On the other hand, the device with BTR‐OH:PC_71_BM as the active layer showed a slightly lower PCE of 8.00%, with a *V*_OC_ of 0.9 V, *J*_SC_ of 13.56 mA cm^−2^, and a FF of 65.3%. By replacing 20% BTR with BTR‐OH in the active layer to form a ternary OSC, the FF was dramatically enhanced from 69.6% to 74.2%, and the *J*_sc_ was boosted from 13.95 mA cm^−2^ to 14.62 mA cm^−2^, leading to the excellent PCE of 10.14% for corresponding ternary device. Both the maximum and average PCEs show ≈12% relative enhancement in the ternary OSC. To the best of our knowledge, the PCE of 10.14% is the record for ternary ASM OSC with dual donors, and among the leading values for all thick‐film ASM OSCs.

The external quantum efficiency (EQE) spectra of the optimized devices were shown in Figure 2b. Compared with the EQE of the binary counterparts, the ternary device exhibited stronger EQE response. Three noticeable features were observed in the EQE spectra. The binary device with BTR‐OH showed narrower spectra that followed its absorption profile—weaker absorption shoulder @630 nm (vs Vironic peak in BTR film). The EQE maxima of BTR‐OH‐ and BTR‐based binary and the ternary blends are 75.28%, 76.25%, and 77.98%, respectively. In addition, higher EQE response of the ternary device was noticed compared with that of the binary device with BTR, indicating more photons were harvested that agreed well with the improved absorption coefficient as mentioned above. In other words, instead of broadening the absorption spectra, the adding of BTR‐OH did enhance the film absorption in the region from 400 to 600 nm, resulting in increased EQE in the region from 400 to 600 nm. The EQE spectra integrated (with the AM 1.5G reference spectrum) *J*_SC_ of the BTR‐OH‐ and BTR‐based binary, and the ternary devices are 13.15, 13.69, and 14.03 mA cm^−2^, respectively, consistent with *J*_SC_ values measured from solar simulator (within 5% error Table 1).

Generally, the FF mainly relies on the balanced hole and electron mobilities as well as efficient charge extraction under biased voltage. Recent researches also showed that FF was largely correlated to the relative rate of both charge recombination and extraction, because both parameters have nonlinearly incremental relationship with the charge carrier mobility.^33^, ^34^, ^35^, ^36^, ^37^, ^38^ The third component with similar chemical structure was designed to optimize the film morphology, thus we now turned to check this parameter in the binary/ternary devices closely by examining the extraction, transport, collection, and recombination behaviors of photogenerated charge carriers.

First, the photocurrent density (*J*_ph_) as a function of the effective voltage (*V*_eff_) was plotted to investigate the charge generation and extraction properties (Figure 2c). The *J*_ph_ = *J*_L_ − *J*_D,_ in which *J*_L_ is the current density under illumination respect to *J*_D_ in dark. The *V*_eff_ = *V*_0_ − *V*_A_, where *V*_0_ is the voltage when *J*_ph_ is equal to 0 and *V*_A_ is the applied bias voltage. At high *V*_eff_ of 2.5 V, all the photogenerated excitons are assumed to be dissociated into free charge carriers and collected by electrodes. Ternary device (BTR:BTR‐OH:PC_71_BM = 0.8: 0.2: 1) presented a saturation current density (*J*_sat_) of 15.03 mA cm^−2^, higher than those of the BTR‐based binary (14.00 mA cm^−2^) and BTR‐OH‐based device (13.83 mA cm^−2^), which agrees with the ternary blend's better light absorption capability. The exciton dissociation efficiency (η_diss_ = *J*_SC_/*J*_sat_) and charge collection efficiency (η_coll_ = *J*_max power_/*J*_sat_) were calculated under the short circuit and maximum power output conditions, respectively. The ternary device revealed a η_diss_ of 98.27% and a η_coll_ of 87.37%, higher than both of BTR‐based (η_diss_ of 97.60% and η_coll_ of 83.70%) and BTR‐OH‐based (η_diss_ of 96.94% and η_coll_ of 78.52%) binary devices, indicating more efficient exciton dissociation and charge collection in the ternary device.

To investigate the charge transport process, both hole and electron carrier mobilities were measured by employing the space‐charge‐limited‐current (SCLC) method. The hole‐only devices were fabricated with the device architectures of ITO/PEDOT:PSS/active layer/spiro‐TPD:CuPc/Au and electron‐only devices with ITO/Al/active layer/LiF/Al. The modified BTR‐OH‐based binary device exhibited much lower hole mobilities, and higher electron mobilities of 1.4 × 10^−5^ and 5.3 × 10^−5^cm^2^ V^−1^ s^−1^ than BTR‐based binary device of 7.3 × 10^−5^ and 2.8 × 10^−5^cm^2^ V^−1^ s^−1^, respectively (Figure 2d and Table S5, Supporting Information). After 20% BTR being replaced by BTR‐OH, almost no sacrifice was noticeable in ternary device with values of 6.8 × 10^−5^ and 2.9 × 10^−5^cm^2^ V^−1^ s^−1^. Furthermore, the more balanced charge mobilities (*µ*_h_/*µ*_e_ = 2.34, Table S5, Supporting Information) were believed to lead to higher FF of 74.2% in the ternary devices.

Although both binary and ternary devices performed well, the FF and *J*_SC_ differences observed reveal that incident photon‐to‐current conversion may not be the only critical factor, and carrier recombination is highly possible to represent a non‐negligible loss channel. To verify the hypothesis, we examined the bimolecular recombination by performing light‐intensity dependence measurements, which examined *J*_SC_ dependence on illumination intensities (Figure 2e).

Previous studies have shown how bimolecular recombination losses in OSCs could be estimated by fitting the *J*_SC_ as a function of incident light intensity data plotted in log scales, taking advantage of the power law equation *J* ∝ I^α^, where α is the power factor.^39^, ^40^ In short, a value of α equal to unity reflects weak/no bimolecular recombination (which means nearly all free carriers are swept out and collected at the electrodes prior to recombination). From fitting the data of *J*_SC_ versus light intensity as illustrated in Figure 2e, we obtain α values of 0.970, 0.939, and 0.977 for BTR‐OH‐based and BTR‐based binary, and ternary devices, respectively, indicating negligible bimolecular recombination losses at short circuit conditions. The low bimolecular recombination losses direct us to analyze another loss mechanism: trap‐assisted Shockley‐Read Hall (SRH) recombination. To detect the trap‐assisted SRH loss, we turned to an examination of the variations of *V*_OC_ as a function of the incident light intensity—data plotted in a natural log scale in Figure 2f; data were fitted to *V*_OC_ ∝ nkT/q ln(*I*), where *k*, *T*, and *q* are the Boltzmann constant, temperature in Kelvin, and the elementary charge, respectively. The parameter *n* (usually in the range of 1 to 2) reflects the presence/absence of carrier traps across the active layer or at interfaces at the semiconductor and electrodes. Any deviations from 1 (trap‐free condition) point to the existence of recombination events and, more specifically, to the existence of trap‐assisted recombination. The fitted data shown in Figure 2f derive a slope value of 1.26 and 1.10 for BTR‐OH‐based and BTR‐based binary devices, suggesting that both binary systems do not suffer notable trap‐assisted recombination. Impressively, the *V*_OC_ versus light intensity data for ternary system obey a slope of *n* = 1.04, indicating even lower trap‐assisted SRH recombination. Overall, these findings imply that the incorporation of a structural similar small molecule donor is capable of suppressing bimolecular recombination and reducing trap‐assisted recombination centers, in line with the much enhanced device performance.

**Figure 3:**
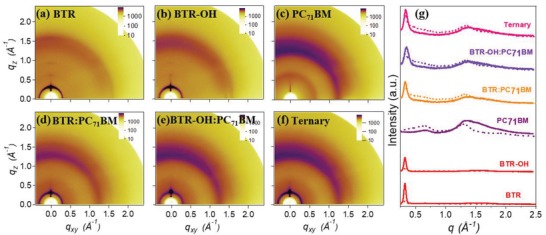
2D GIWAXS patterns of a) BTR, b) BTR‐OH, c) PC_71_BM neat films and d) BTR:PC_71_BM, e) BTR‐OH:PC_71_BM, f) ternary blend films. g) The corresponding GIWAXS intensity profiles along the in‐plane (dashed lines) and out‐of‐plane (solid lines) directions.

Grazing‐incidence wide‐angle X‐ray scattering (GIWAXS) was adopted to investigate molecular packing and orientation in thick films (**Figure** **3**), and the corresponding GIWAXS parameters were concluded in Table S4 in the Supporting Information. BTR and BTR‐OH demonstrated similar molecular packing and orientation, which were composed of strong edge‐on orientation with weak face‐on orientation in Figure 3a,b. The coexistence of edge‐on and face‐on orientations are capable of potentially forming a 3D network for charge hopping and finally result in the pronounced enhancement of charge transport.^31^, ^41^, ^42^ The optimized BTR‐based binary blend film presented two very strong peaks from the lamellar (100) diffraction at q ≈ 0.33 Å^−1^ with crystal coherence length (CCL) of 13.46 nm for in‐plane (IP) direction, and q ≈ 0.33 Å^−1^ with CCL of 9.12 nm for out‐of‐plane (OOP) direction (Figure 3d,g, Table S4, Supporting Information), which means extremely strong crystallinity was demonstrated. In contrast, the optimized BTR‐OH‐based binary blend film exhibited two relatively weak peaks from the lamellar (100) diffraction at q ≈ 0.34 Å^−1^ with CCL of 8.31 nm for IP direction and q ≈ 0.35 Å^−1^ with CCL of 7.07 nm for OOP direction (Figure 3e,g, Table S4, Supporting Information), which indicate relatively low crystallinity was presented due to the terminal group (TG) modification for the small molecule donor. When 20% BTR was replaced by BTR‐OH in the optimized ternary film, the crystallinity of the ternary blend film was impressively tuned with two medium peaks from the lamellar (100) diffraction at q ≈ 0.33 Å^−1^ with CCL of 12.29 nm for IP direction and q ≈ 0.34 Å^−1^ with CCL of 8.19 nm for out‐of‐plane(OOP) direction (Figure 3f,g, Table S4, Supporting Information).

Thus, TG modification is proved to be effective to tune the crystallinity of the small molecule donor. Furthermore, BTR‐based binary blend presented exceedingly strong crystallinity, which was attributed to excessive phase separation and large length scale donor–acceptor (D–A) networks. Replacing 20% BTR with lower crystallinity small molecule donor BTR‐OH could dramatically improve the morphology and tune the crystallinity of the blend, and finally lead to the obvious enhancement of the device performance (Table 1).

**Figure 4:**
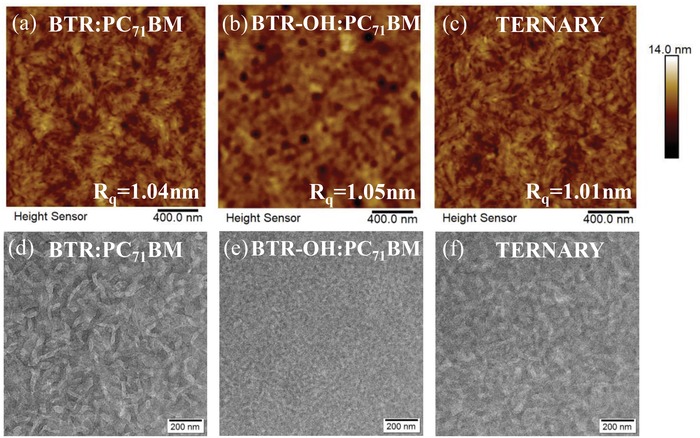
AFM height images a–c) and TEM images d–f) of a,d) BTR:PC_71_BM binary, b,e) BTR‐OH:PC_71_BM binary and c,f) BTR:BTR‐OH:PC_71_BM(0.8:0.2:1 w/w) ternary blends.

In order to verify the above hypothesis, atomic force microscopy (AFM) and transmission electron microscopy (TEM) were used to study the phase separation shown in **Figure** **4**a–f. As presented in Figure S4 in the Supporting Information, AFM height images of BTR and BTR‐OH neat film presented root mean square roughness (Rq) of 3.91 and 2.49 nm, implying TG modification sharply decreases the aggregation of the small molecule donor, which was consistent with the reduced crystallinity in Table S4 in the Supporting Information. In addition, the introduction of PC_71_BM further decreases the aggregation, resulting in the Rq of BTR:PC_71_BM (1.04 nm), BTR‐OH:PC_71_BM (1.03 nm), and BTR:BTR‐OH:PC_71_BM (1.01 nm) (Figure 4a–c). Theoretically, smaller aggregated domains lead to larger donor/acceptor interfacial area which dramatically contributes to exciton dissociation, and the interpenetrating network facilitates both hole and electron transport.^43^, ^44^, ^45^, ^46^, ^47^, ^48^, ^49^, ^50^ Therefore, the uniform and smooth films with reduced aggregated domains in ternary blend are favorable for interface contact and charge collection, which in line with the photovoltaic performance in Table 1.

TEM images (Figure 4d,e) clearly displayed the distinct donor–acceptor networks of different length scales. More phase‐separated D–A networks but weak crystallinity (confirmed by GIWAXS, Table S4, Supporting Information) for BTR‐OH‐based binary films result in the reduced PCE. Meanwhile, less phase‐separated D–A networks with strong crystallinity for BTR‐based binary films also lead to relatively weak device performance. However, the phase‐separated D–A networks (Figure 4f) and crystallinity of the blends were dramatically optimized leading to suitable domain size of ≈20 nm after the replacement of BTR with BTR‐OH, which concurrently yield high device PCE (>10%). Therefore, TG strategy is capable of simultaneously tuning the crystallinity and optimizing the phase aggregation of the small molecule donor. It is worth noting that adding a structurally similar third component without absorption broadening is demonstrated to be a simple and effective approach to fine‐tune the optimum BHJ morphology toward to high performance thick‐film ASM OSCs in this contribution.

At last, we noticed that the photoluminescence (PL) of BTR could be partially quenched by BTR‐OH in the BTR:BTR‐OH blend films with different BTR‐OH loadings as presented in Figure S3 in the Supporting Information, indicating that there may be charge transfer between the two donor materials. To verify this, a series of devices were fabricated with BTR and BTR‐OH in neat and blends with different weight ratios as active layer (Figure S2, Supporting Information). The BTR and BTR‐OH neat film as well as blend films with different weight ratios presented nearly identical *J*_sc_ values around 0.01 mA cm^−2^, confirming that charge transfer between the two small molecule donors is negligible.

## 3 Conclusions

In summary, we designed and synthesized a dehydroxyated derivative of BTR, namely BTR‐OH, which has similar absorption profiles with BTR, and utilized BTR‐OH to construct ternary OSCs based on BTR:BTR‐OH:PC_71_BM system. Compared with the binary counterparts, the BTR:BTR‐OH:PC_71_BM ternary device demonstrated the champion PCE of 10.14% and FF of 74.2% with a film thickness of ≈300 nm at a weight ratio of 0.8:0.2:1, which was among the leading values for thick‐film ASM OSCs. BTR‐OH plays a versatile role in ternary OSCs: 1) BTR‐OH enhanced absorption of photoactive layer, which contributed to the *J*_SC_; 2) BTR‐OH suppressed bimolecular/trap‐assisted recombination, and therefore improved charge extraction; 3) BTR‐OH decreased the crystallinity of the donor phase, and optimized the phase‐separated D/A network and domain size to a suitable scale, thereby balancing the hole and electron mobilities. Our findings demonstrated that the donor derivative with similar chemical structure and absorption profile but different crystallinity feature can be a promising third component strategy for constructing high‐performance ternary ASM OSC device.

## Conflict of Interest

The authors declare no conflict of interest.

## Supporting information

Supplementary
