# Supplementary material for: Donor Derivative Incorporation: An Effective Strategy toward High Performance All‐Small‐Molecule Ternary Organic Solar Cells
Source: Adv Sci (Weinh). 2019 Sep 4;6(21):1901613. doi: 10.1002/advs.201901613 (PMC6839630; doi:10.1002/advs.201901613)
Supplement: Supplementary file 1 — Supplementary [file ADVS-6-1901613-s001.pdf]

Copyright WILEY-VCH Verlag GmbH & Co. KGaA, 69469 Weinheim, Germany, 2019.

ADVANCED SCIENCE
Open Access

# Supporting Information

for *Adv. Sci.,* DOI: 10.1002/advs.201901613

# Donor Derivative Incorporation: An Effective Strategy toward High Performance All-Small-Molecule Ternary Organic Solar Cells

*Hua Tang, Tongle Xu, Cenqi Yan, Jie Gao, Hang Yin, Jie Lv, Ranbir Singh, Manish Kumar, Tainan Duan, Zhipeng Kan,* Shirong Lu,* and Gang Li**

WILEY-VCH

Copyright WILEY-VCH Verlag GmbH & Co. KGaA, 69469 Weinheim, Germany, 2019.

Supporting Information

# Donor Derivative Incorporation – An Effective Strategy Towards High Performance All-Small-Molecule Ternary Organic Solar Cells

Hua Tang, Tongle Xu, Cenqi Yan, Jie Gao, Hang Yin, Jie Lv, Ranbir Singh, Manish Kumar, Tainan Duan, Zhipeng Kan, * Shirong Lu, * and Gang Li *

H. Tang, T. Xu, J. Gao, J. Lv, Dr T. Duan, Dr Z. Kan, Dr S. Lu
Chongqing Institute of Green and Intelligent Technology
Chinese Academy of Sciences, Chongqing, 400714, China
E-mail: kanzhipeng@cigit.ac.cn, lushirong@cigit.ac.cn
H. Tang, T. Xu, J. Lv
University of Chinese Academy of Sciences
Beijing 100049, China
H. Tang, Dr C. Yan, Dr H. Yin, Dr G. Li
Department of Electronic and Information Engineering, The Hong Kong Polytechnic University, Hong Hum, Kowloon, Hong Kong, China.
E-mail: gang.w.li@polyu.edu.hk
Dr. R. Singh
Department of Energy & Materials Engineering, Dongguk University, Seoul, Republic of Korea
Dr. M. Kumar
Pohang Accelerator Laboratory, Pohang University of Science and Technology, Pohang, Republic of Korea.

**Content**

1. General Experimental Details ........................................................................3

2. Synthesis of BTR-OH and Characterizations ..............................................................4

3. Cyclic voltammograms........................................................................................5

4. Device Fabrication ..........................................................................................6

5. Additional PV Device Performance Data........................................................................7

6. Photoluminescence (PL) Quenching .............................................................................9

7. Atomic Force Microscopy (AFM) Imaging.......................................................................10

8. Transmission Electron Microscopy (TEM) Characterization......................................11

9. Grazing Incidence Wide-angle X-ray Scattering .........................................................11

10. SCLC Measurements.........................................................................................12

11. Solution NMR Spectra.......................................................................................13

12. Mass Spectra ..............................................................................................15

## 1. General Experimental Details

All reactions were performed under nitrogen atmosphere and solvents were purified and dried from appropriate drying agents using standard techniques prior to use. Reagents available from commercial sources were used without further purification unless otherwise stated. Flash chromatography was performed by using Silicycle Silica Flash P60 (particle size 40-63 μm, 60 Å, 230-400 mesh) silica gel. Silica gel on TLC-PET foils from Fluka was used for TLC. Precursor 1 and 2 were prepared using literature methods.[1-3] All compounds were characterized by NMR spectroscopy on Bruker Avance III Ultrashield Plus instruments (600 MHz). The spectra were referenced on the internal standard TMS. High-resolution mass spectrometry (HRMS) data was recorded using a Thermo Scientific-LTQ Velos Orbitrap MS. Elemental analyses were obtained commercially through Chemical & Analytical Services Pty Ltd. Note: Spectroscopy-grade $CHCl_3$ was filtered through basic alumina prior to use in order to suppress solvent acidity and avoid undesired protonation reactions that may influence the spectral absorption of the molecular acceptors described in this study.

## 2. Synthesis of BTR-OH and Characterizations

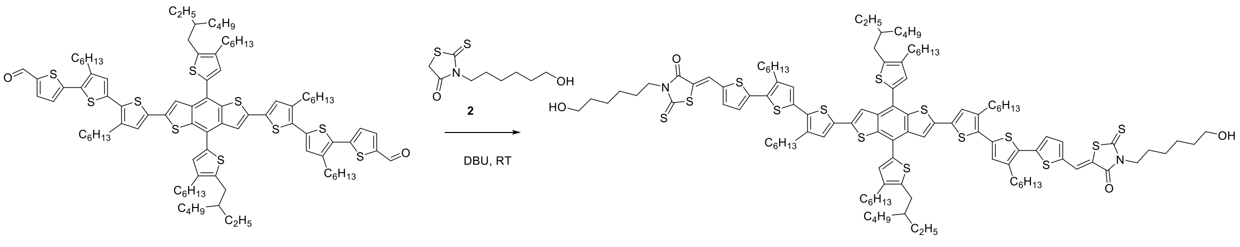


1

Compound 1 (0.5 g, 0.3 mmol) was dissolved in a dry chloroform (10 mL) and few drops of triethylamine was added under nitrogen. Then, compound 2 (0.3 g, 1.3 mmol) was added and resulting solution was heated to reflux and stirred for 12 hours. The reaction mixture was cooled down to room temperature, poured into methanol. The precipitate was filtered and washed several times with methanol. After purified by silica gel column chromatography (eluent: CH2Cl2/MeOH = 98/2, v/v), a dark black solid BTR-OH (0.6 g, 94%) was obtained.

$^{1}$H NMR (600 MHz, CDCl3) δ 7.82 (s, 2H), 7.64 (s, 2H), 7.34 (d, J = 3.6 Hz, 2H), 7.23 (s, 2H), 7.19 (d, J = 3.0 Hz, 2H), 7.09 (s, 2H), 6.99 (s, 2H), 4.10 (t, J = 7.2 Hz, 4H), 3.64 (t, J = 6.0 Hz, 4H), 2. 83– 2.75 (m, 12H), 2.66 (t, J = 7.2 Hz, 4H), 1.77– 1.66 (m, 20H), 1.47 – 1.32 (m, 62H), 1.00 – 0.89 (m, 30H).$^{13}$C NMR (150 MHz, CDCl3) δ 191.15, 166.50, 143.33, 140.92, 140.02, 138.49, 137.96, 137.58, 136.24, 136.02, 134.72, 134.67, 133.89, 133.61, 129.44, 128.84, 128.67, 128.00, 127.25, 125.50, 124.02, 122.46, 119.13, 118.44, 61.76, 43.62, 40.73, 31.75, 31.56, 31.43, 30.86, 30.70, 30.66, 29.78, 29.33, 29.28, 28.88, 28.73, 28.31, 28.28, 28.22, 28.00, 27.49, 25.90, 25.46, 25.11, 24.22, 22.11, 21.71, 21.64, 21.61, 13.21, 13.15, 13.10, 13.08, 10.06. MS (MALDI) m/z 2,063.793 $[M]^{+}$. Elemental analysis found: C, 66.296; H, 6.744; N, 1.312; O, 3.419; S, 22.407

## 3. Cyclic voltammograms

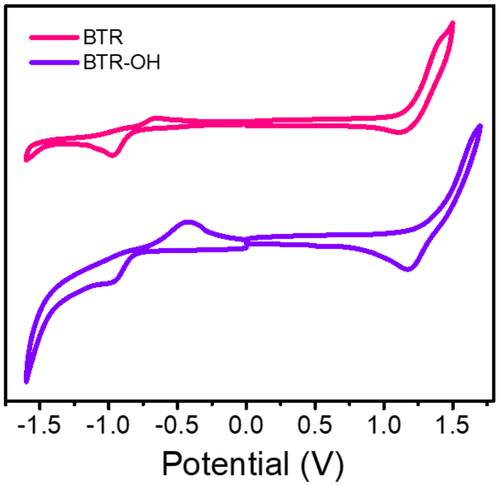


**Figure S1**. Cyclic voltammograms for SM donors BTR and BTR-OH.

## 4. Device Fabrication

The all-small-molecule organic solar cells were prepared on glass substrates with tin-doped indium oxide (ITO, 15 Ω/sq) patterned on the surface (device area: 0.08 $cm^2$). Substrates were prewashed with isopropanol to remove organic residues before immersing in an ultrasonic bath of soap for 15 min. Samples were rinsed in flowing deionized water for 5 min before being sonicated for 15 min each in successive baths of deionized water, acetone and isopropanol. Next, the samples were dried with pressurized nitrogen before being exposed to a UV-ozone plasma for 20 min. A thin layer of PEDOT:PSS (~30nm) (Clevios AL4083) was spin-coated onto the UV-treated substrates, the PEDOT-coated substrates were subsequently annealed on a hot plate at 150 °C for 20 min, and the substrates were then transferred into the glovebox for active layer deposition.

All solutions were prepared in the glovebox using the SM donors (BTR or BTR-OH) and the SM acceptor $PC_{71}BM$; the SM donor BTR-OH was synthesized as mentioned above. The BTR was purchased from 1 Material Tech Inc., and the $PC_{71}BM$ was purchased from lumtech Inc. Optimized devices were obtained by dissolving BTR, BTR-OH and $PC_{71}BM$ in chloroform (CF) using a D/A ratio of 1:0:1, 0:1:1 and 0.8:0.2:1 (wt/wt), total concentration of 40mg/ml. Note: The as-prepared solutions were stirred for 3 hours at room temperature before being spin coat on the PEDOT:PSS substrates. The active layers were spin-coated at an optimized speed of 900~1200 rpm for time period of 45s, resulting in films of 250 to 300 nm in thickness. The active layers were then exposed to solvent vapor annealing (SVA) with Dichloromethane (DCM) vapors for 10-55s.

Then, a ~10nm-thin layer of Phen-NaDPO was coated on top as electron transport layer after SVA treatment. The samples were then dried at room temperature for 1 hour. Next, the samples were placed in a thermal evaporator for evaporation of a 90 nm-thick layer of Silver (Ag) evaporated at 1.5 Å $s^{-1}$; pressure of less than $2x10^{-6}$ Torr. Following electrode deposition, samples underwent J−V testing.

The current density-voltage (J-V) curves of devices were measured using a Keithley 2400 Source Meter in glove box under AM 1.5G (100 mW $cm^{-2}$) using a Enlitech solar simulator. A 2×2 $cm^2$ monocrystalline silicon reference cell with KG5 filter (purchased from Enli Tech. Co., Ltd., Taiwan). The EQE spectra were measured using a Solar Cell Spectral Response Measurement System QE-R3011 (Enlitech Co., Ltd.). The light intensity at each wavelength was calibrated using a standard monocrystalline Si photovoltaic cell.

## 5. Additional PV Device Performance Data

**Table S1**. Summary of average PV performance for BTR:BTR-OH:$PC_{71}BM$ ternary active layers cast from CF, and subjected to various D/A ratio.

| BTR:BTR-OH: $PC_{71}BM$ | $V_{OC}$ [V] | $J_{SC}$ [mA cm$^{-2}$] | *FF* [%] | Avg. PCE [%] | Max. PCE [%] |
|---|---|---|---|---|---|
| 0.9:0.1:1 | 0.94 | 13.75 | 72.84 | 9.14 | 9.38 |
| 0.7:0.3:1 | 0.92 | 14.11 | 72.49 | 9.22 | 9.45 |
| 0.5:0.5:1 | 0.92 | 13.92 | 71.15 | 8.94 | 9.10 |

**Table S2**. Summary of average PV performance for BTR:BTR-OH:$PC_{71}BM$ ternary devices with different active layer thickness..

| Device | Thickness [nm] | $V_{OC}$ [V] | $J_{SC}$ [mA cm$^{-2}$] | *FF* [%] | Avg. PCE [%] | Max. PCE [%] |
|---|---|---|---|---|---|---|
| Ternary 0.8:0.2:1 | *ca.* 80 | 0.93 | 11.09 | 75.6 | 7.55 | 7.80 |
| | *ca.* 150 | 0.94 | 12.23 | 76.6 | 8.58 | 8.81 |
| | *ca.* 200 | 0.93 | 13.55 | 73.4 | 9.21 | 9.30 |
| | *ca.* 250 | 0.93 | 14.58 | 73.2 | 9.76 | 10.03 |
| | *ca.* 300 | 0.93 | 14.62 | 74.2 | 9.98 | 10.14 |
| | *ca.* 400 | 0.92 | 14.71 | 69.9 | 9.23 | 9.44 |
| | *ca.* 500 | 0.92 | 14.62 | 64.0 | 8.31 | 8.58 |

**Table S3**. Summary of average PV performance for BTR-OH:$PC_{71}BM$ binary active layers cast from CF, and subjected to various condition.

| Condition | SVA (THF) [s] | $V_{OC}$ [V] | $J_{SC}$ [mA cm$^{-2}$] | *FF* [%] | Avg. PCE [%] | Max. PCE [%] |
|---|---|---|---|---|---|---|
| D:A=1:1 20mg/ml | 0 | 0.97 | 10.67 | 40.32 | 4.05 | 4.17 |
| | 5 | 0.94 | 10.25 | 62.37 | 5.81 | 6.00 |
| | 10 | 0.90 | 9.00 | 52.05 | 4.12 | 4.21 |

| | | | | | | |
|---|---|---|---|---|---|---|
| D:A=1:1 30mg/ml | 0 | 0.95 | 9.44 | 36.00 | 3.03 | 3.22 |
| | 5 | 0.94 | 10.28 | 60.45 | 5.65 | 5.83 |
| | 10 | 0.92 | 9.09 | 57.76 | 4.66 | 4.82 |
| D:A=6:4 20mg/ml | 0 | 0.95 | 11.22 | 47.43 | 4.87 | 5.05 |
| | 5 | 0.94 | 9.29 | 52.33 | 4.28 | 4.56 |
| | 10 | 0.90 | 8.16 | 45.28 | 2.96 | 3.32 |
| D:A=4:6 20mg/ml | 0 | 0.90 | 9.32 | 37.46 | 3.02 | 3.13 |
| | 5 | 0.83 | 9.46 | 54.35 | 4.09 | 4.26 |
| | 10 | 0.83 | 9.90 | 49.01 | 3.88 | 4.01 |
| D:A=1:1 40mg/ml | 0 | 0.96 | 12.56 | 41.41 | 4.80 | 4.98 |
| | 5 | 0.94 | 11.67 | 69.24 | 7.33 | 7.57 |
| | 10 | 0.76 | 10.63 | 55.54 | 4.32 | 4.47 |

| Condition | SVA (DCM) [s] | $V_{OC}$ [V] | $J_{SC}$ [mA cm$^{-2}$] | *FF* [%] | Avg. PCE [%] | Max. PCE [%] |
|---|---|---|---|---|---|---|
| D:A=1:1 40mg/ml | 10 | 0.89 | 10.81 | 59.88 | 5.58 | 5.74 |
| | 25 | 0.89 | 12.10 | 62.08 | 6.51 | 6.69 |
| | 40 | 0.90 | 13.56 | 65.31 | 7.85 | 8.00 |
| | 55 | 0.90 | 12.90 | 66.47 | 7.53 | 7.74 |

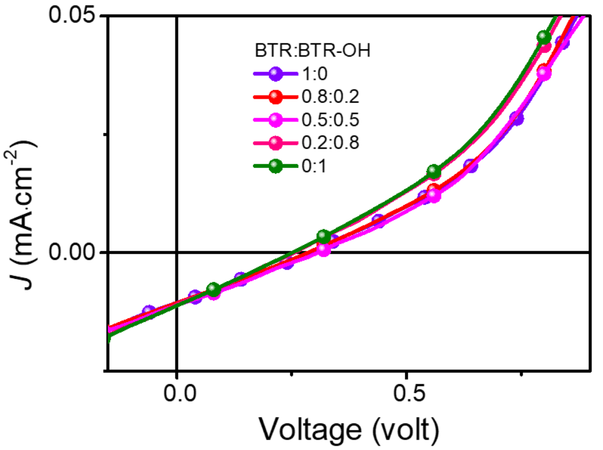


**Figure S2**. The current density-voltage (J-V) characteristics of organic cells based on BTR, BTR-OH and BTR: BTR-OH blend films with different BTR-OH contents under simulated AM 1.5G irradiation (100mW cm$^{-2}$)

## 6. Photoluminescence (PL) Quenching

Samples for PL spectroscopy were spin-coated onto glass substrates subject to various conditions. Spectra were measured using a spectrofluorometer FluoroMax-4, HORIBA.

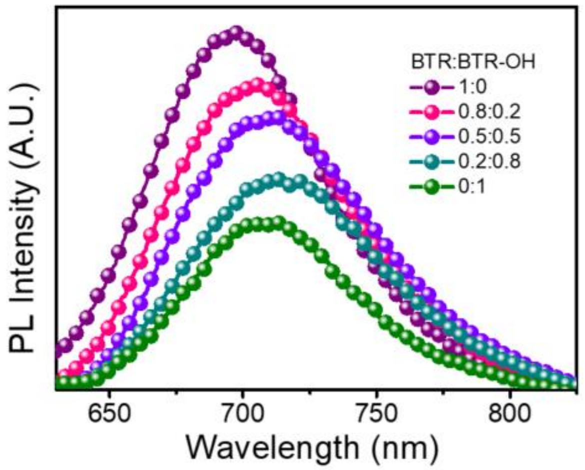


**Figure S3**. Photoluminescence (PL) spectra of BTR:BTR-OH blend films with different BTR-OH contents.(Excited at 595nm)

## 7. Atomic Force Microscopy (AFM) Imaging

A Dimension Icon atomic force microscope (AFM) from Bruker was used to image the active layers in tapping mode (heights and phase images are represented below).

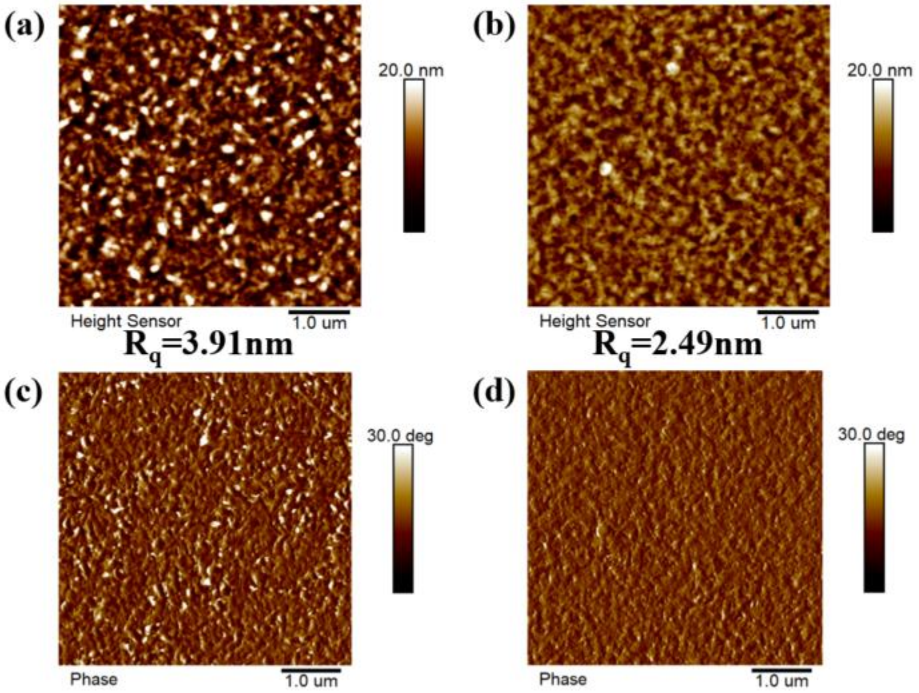


**Figure S4**. AFM images (5x5 μm$^2$) of (a, b) topography and (c,d) phase mode for **BTR** and **BTR-OH** neat films. (a, c) Neat film of **BTR**, RMS roughness: 3.91nm; (b, d) Neat film of **BTR-OH**, RMS roughness: 2.49nm.

## 8. Transmission Electron Microscopy (TEM) Characterization

Films were spun-cast on PEDOT:PSS-coated glass substrates. The BTR-based and BTR-OH-based binary and ternary BHJ films were floated off the substrates in deionized water and collected on lacey carbon coated TEM grids (Electron Microscopy Sciences). TEM studies were performed a Thermo Fischer (former FEI) Titan Titan 80-300 TEM equipped with an electron monochromator and a Gatan Imaging Filter (GIF) Quantum 966.

## 9. Grazing Incidence Wide-angle X-ray Scattering

Silicon substrates for GIWAXS test were sonicated for 15 min each in successive baths of detergent, DI water, acetone and isopropanol. The substrates were then dried with pressurized nitrogen before being exposed to the UV−ozone plasma for 20 min. The BHJ layers were prepared following methods described in Section of Device Fabrication. GIWAXS measurements were carried out at 5A beamline of the Pohang Light Source II (PLS-II) in South Korea. The GIWAX images were recorded at 0.13 incidence angle with X-rays of 11.57 keV (λ=1.0716Å) and MAR345 image plate detector.

**Table S4.** Detailed GIWAXS (100) peak information IP and OOP of BTR- and BTR-OH-based neat, binary and ternary films.

| Component | Peak | Peak location (Å$^{-1}$) | FWHM (Å−1) | Crystal coherence length(nm) |
|---|---|---|---|---|
| BTR | (100)IP | 0.32 | 0.033 | 17.13 |
| | (100)OOP | 0.32 | 0.040 | 14.13 |
| BTR-OH | (100)IP | 0.32 | 0.047 | 12.03 |
| | (100)OOP | 0.32 | 0.043 | 13.14 |
| BTR:$PC_{71}BM$ | (100)IP | 0.33 | 0.042 | 13.46 |
| | (100)OOP | 0.33 | 0.062 | 9.12 |
| BTR-OH:$PC_{71}BM$ | (100)IP | 0.34 | 0.068 | 8.31 |
| | (100)OOP | 0.35 | 0.080 | 7.07 |
| Ternary | (100)IP | 0.33 | 0.046 | 12.29 |
| | (100)OOP | 0.34 | 0.069 | 8.19 |

## 10. SCLC Measurements

Hole-only devices were fabricated for the J-V measurements. The device structure was ITO/PEDOT:PSS/BHJ/CuPc:spiro-TPD/Au. The PEDOT:PSS layer was the hole injection layer, and the electron blocking layer (EBT) was a mixed layer of spiro-TPD and CuPc. The single-carrier device was connected to a Source Measure Unit (Keithley, Model 236 SMU), which provided DC voltage to the hole-only devices. The SMU was also used to record the currents under different DC voltage conditions. The J-V signals was detected and recorded. The J-V characteristics were further analyzed by the space-charge-limited-current (SCLC) method to extract zero-field carrier mobilities, where SCLC is described by:

$$J = \frac{9\varepsilon_0\varepsilon_\gamma\mu_0 V^2}{8L^3}\exp\left(0.89\beta\sqrt{\frac{V}{L}}\right)$$

where J is the current density, L is the film thickness of the active layer, $\mu_0$ is the hole or electron mobility, $\varepsilon_r$ is the relative dielectric constant of the transport medium, $\varepsilon_0$ is the permittivity of free space ($8.85 \times 10^{-12}$ F m$^{-1}$), V (= $V_{appl}$ - $V_{bi}$) is the internal voltage in the device, where $V_{appl}$ is the applied voltage to the device and $V_{bi}$ is the built-in voltage due to the relative work function difference of the two electrodes.

**Table S5**. Summary of carrier mobilities.

| BTR:BTR-OH:$PC_{71}BM$ | $\mu_h$ (cm$^2$ V$^{-1}$ s$^{-1}$) | $\mu_e$ (cm$^2$ V$^{-1}$ s$^{-1}$) | $\mu_h/\mu_e$ |
|---|---|---|---|
| 1:0:1 | $7.3\times10^{-5}$ | $2.8\times10^{-5}$ | 2.61 |
| 0:1:1 | $1.4\times10^{-5}$ | $5.3\times10^{-5}$ | 0.26 |
| 0.8:0.2:1 | $6.8\times10^{-5}$ | $2.9\times10^{-5}$ | 2.34 |

## 11. Solution NMR Spectra

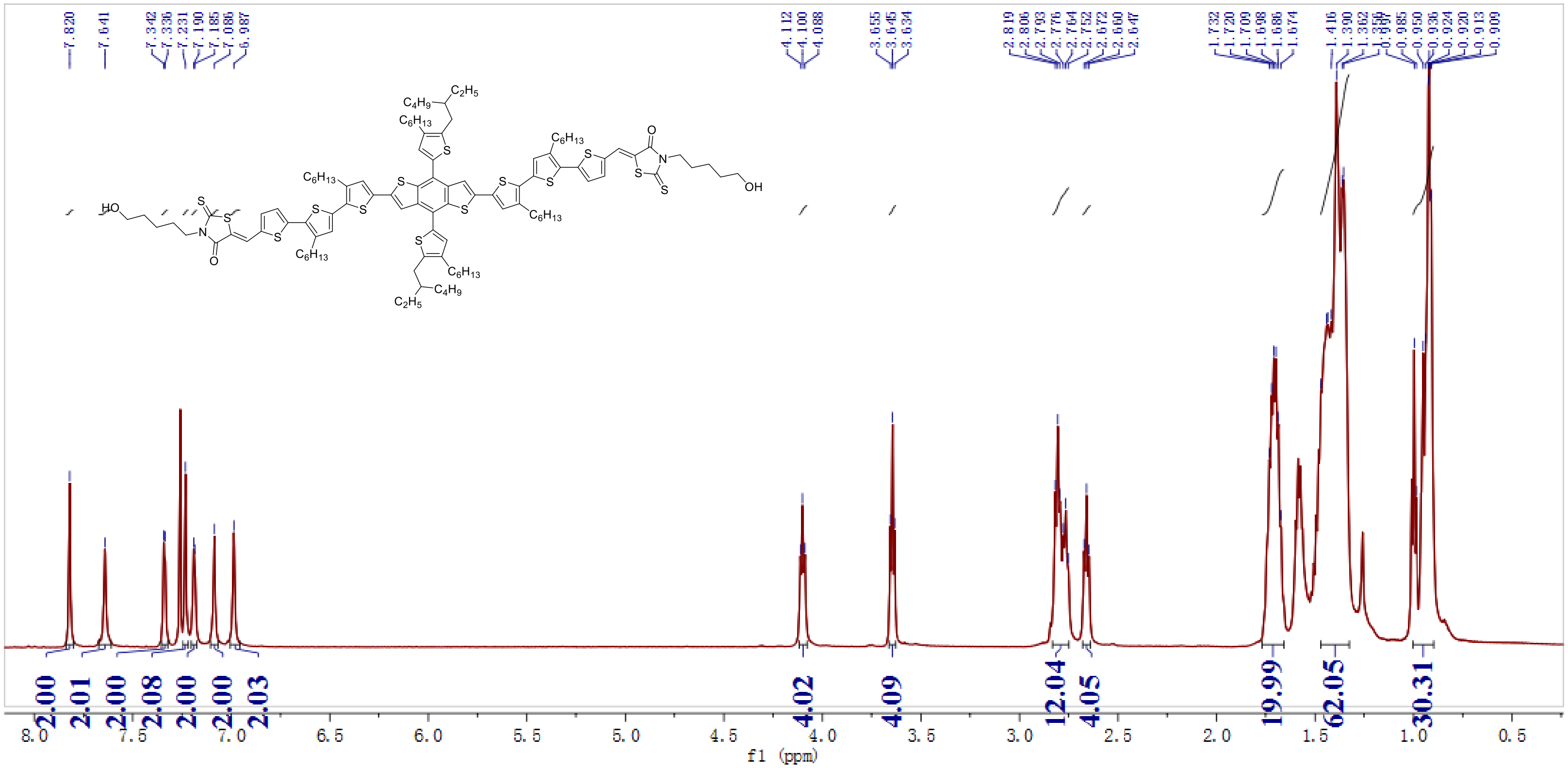

**Figure S5.** $^{1}$H NMR spectrum of **BTR-OH** in $CDCl_3$.

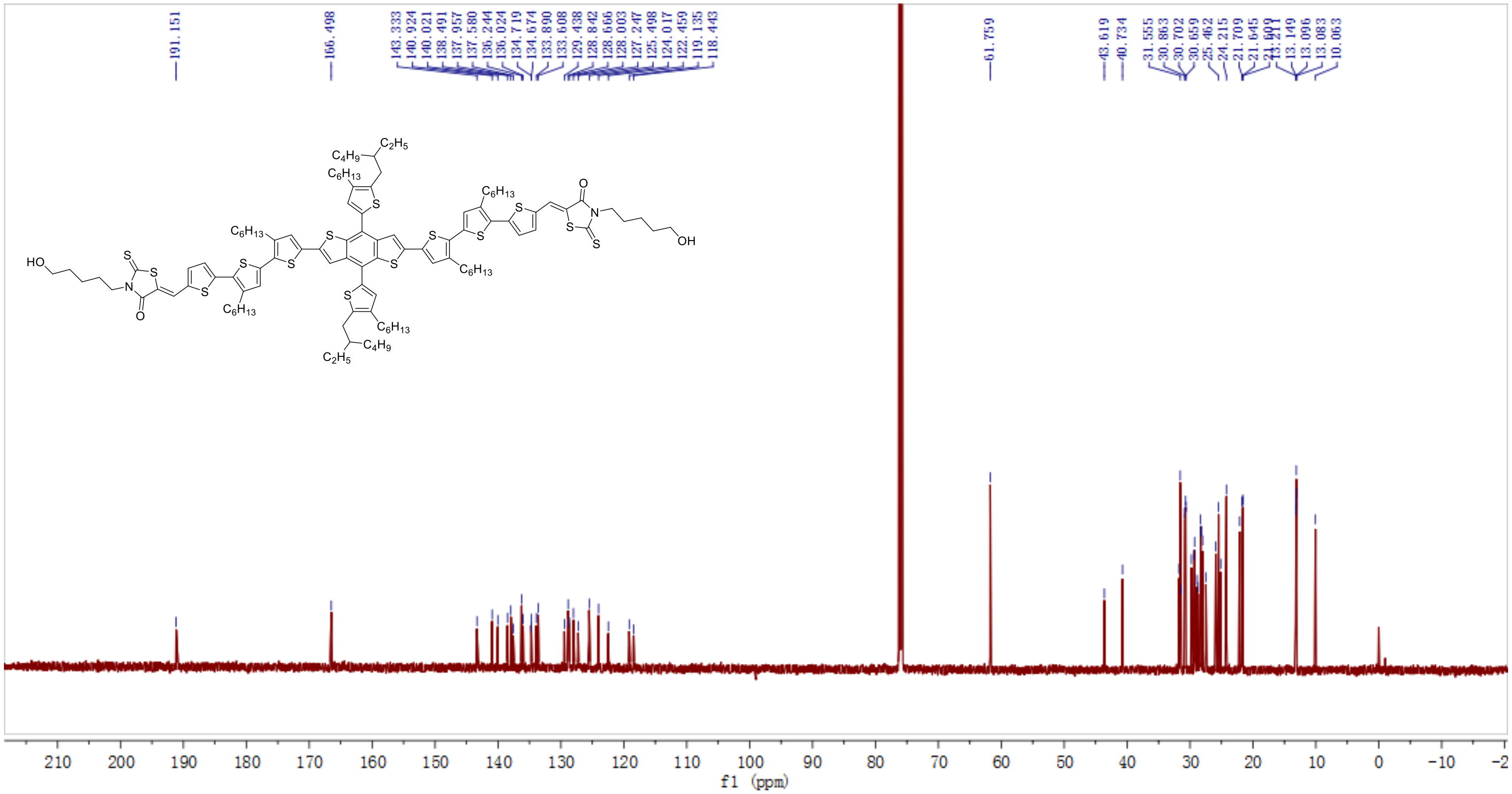


**Figure S6.** $^{13}$C NMR spectrum of **BTR-OH** in CDCl

## 12. Mass Spectra

# Mass Spectrum List Report

**Analysis Info**

| | | | |
|---|---|---|---|
| Analysis Name | D:\Data\FJH\FJH_0_B1_000027.d | Acquisition Date | 8/2/2019 10:13:19 PM |
| Method | 5-12-DADOU | Operator | |
| Sample Name | 2018-6-7-MD | Instrument | solariX |
| Comment | PeptideMix NS=8 TF=1.2 | | |

**Acquisition Parameter**

| | | | | | |
|---|---|---|---|---|---|
| Polarity | Positive | n/a | n/a | No. of Laser Shots | 100 |
| n/a | n/a | No. of Cell Fills | 1 | Laser Power | 70.0 lp |
| Broadband Low Mass | 53.8 m/z | n/a | n/a | n/a | n/a |
| Broadband High Mass | 2500.0 m/z | n/a | n/a | n/a | n/a |
| Acquisition Mode | Single MS | n/a | n/a | | |
| Pulse Program | basic | n/a | n/a | Calibration Date | Fri Feb 21 02:36:54 2014 |
| Source Accumulation | 0.100 sec | n/a | n/a | Data Acquisition Size | 2097152 |
| Ion Accumulation Time | 0.500 sec | n/a | n/a | Apodization | Sine-Bell Multiplication |
| Flight Time to Acq. Cell | 0.001 sec | n/a | n/a | Apodization | Apodization |

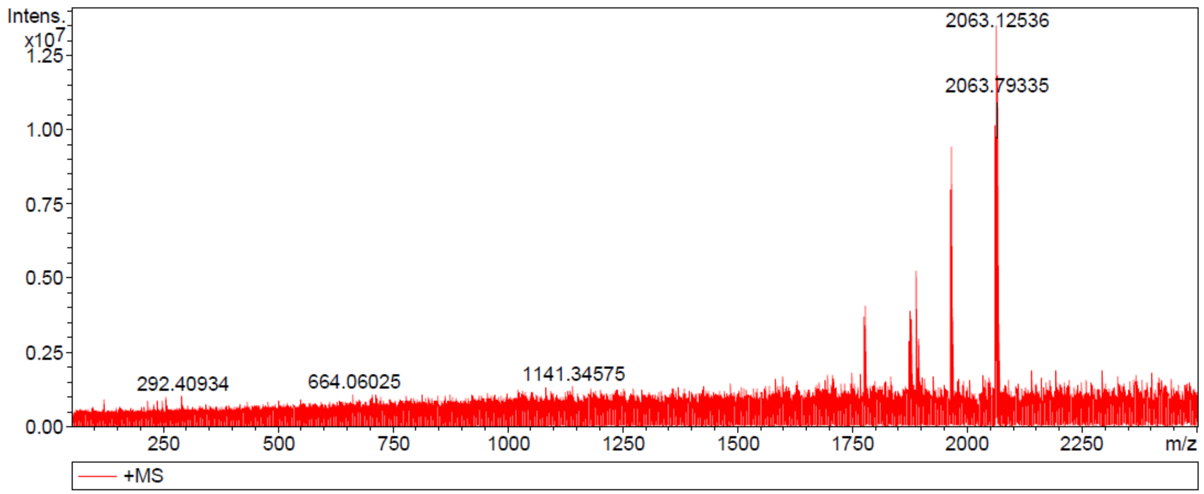

[1] K. Sun, Z. Xiao, S. Lu, W. Zajaczkowski, W. Pisula, E. Hanssen, J. M. White, R. M. Williamson, J. Subbiah, J. Ouyang, A. B. Holmes, W. W. Wong, D. J. Jones, *Nat. Commun.* **2015**, *6*, 6013.

[2] C. Nitsche, C. D. Klein, *Tetrahedron Letters* **2012**, *53*, 5197.

[3] T. L. Nguyen, T. H. Lee, B. Gautam, S. Y. Park, K. Gundogdu, J. Y. Kim, H. Y. Woo, *Adv. Funct. Mater*. **2017**, *27*, 1702474.
